# Three dimensional ultrastructure of human respiratory cilia in health and disease

**DOI:** 10.1186/2046-2530-1-S1-P47

**Published:** 2012-11-16

**Authors:** A Shoemark, T Burgoyne, M Dixon, P Luther, C Hogg

**Affiliations:** 1 Department of Paediatric Respiratory Medicine, Royal Brompton and Harefield NHS Trust, UK; 2 National Heart and Lung Institute, Imperial College London, UK

## 

Motile cilia in the respiratory tract beat to clear mucus from the airways, keeping the lung clean and free from infection. In the inherited genetic condition Primary Ciliary Dyskinesia (PCD) cilia beat is ineffective, resulting in chronic cough, recurrent chest infections and rhinosinustitis. The definitive diagnostic test for PCD is electron microscopy of ciliary ultrastructure. There are a number of known ultrastructural defects associated with PCD. We have recently established the first three dimensional model of human respiratory cilia ultrastructure using electron tomography. Electron tomography is an electron microscopy technique which allows increased resolution and visualisation of structures in three dimensions. Our model highlights key features of the axoneme which cannot be resolved using traditional electron microscopy. The aim of this study was to characterise in 3D the ultrastructure of defects associated with PCD using the normal model as a template. Tomograms of transverse and longitudinal sections of cilia were generated from nasal brush biopsies taken from patients with PCD. Key features of the cilium were resolved using sub- volume or sub-tomographic averaging and measured. We show in detail the ultrastructural phenotype of radial spoke, dynein arm and central pair defects allowing insight into the structure of human respiratory tract cilium and primary ciliary dyskinesia.

